# Professional Liberty and Restraint

**Published:** 1866-01

**Authors:** 


### PROFESSIONAL LIBERTY AND RESTRAINT.

Every one has the right and should have the liberty, to do that which he can do well, provided he does not operate injury to any, but good to the largest possible number.

This rule strictly applied, would bring about us a different state of affairs from that which at present exists. In some things it is admissable for one to do that which he cannot well accomplish, but in *some* only. There are somethings in which none should be permitted to engage who are not competent to produce good results.

And certainly, in nothing can this be more certainly true, than of those who would assume to be in any particular the conservators of the health of their fellow beings.

In this country a liberty quite incompatible with the health and welfare of humanity is granted. Any one, with no preparation at all, or with the most false, and erroneous teachings, may go forth and assume the duties and responsibilities of the physician, in either general or particular practice, without let or hindrance, so far as legal barriers are concerned, at least so long as his operations do not result in palpable manslaughter.

This state of affairs exists to an alarming extent in the dental department of the healing art, though perhaps not more so than in some other specialties, or than in general practice, but in the dental profession it is more palpable to us.

Why he who knows nothing of the teeth, nothing of the care requisite for their preservation, less of the diseases to which they are subjected, and still less of the treatment necessary for their restoration, and still less of the multitudinous complications that modify these things, should be permitted to assume the duties and responsibilities of the dentist, we are unable to conceive. Ruin and death, of these precious organs of the human economy, the teeth, follow right along in the wake of such miserable pretenders. The health, and even the life, depends largely upon the teeth, and he who trifles with the teeth, trifles with these. No one should be permitted to perform the functions of a dentist, without the proper qualifications. But the people are not able to distinguish between him who is qualified, and him who is not, or at least till they have purchased this ability, by a sacrifice too grievous to be borne.

In the more ordinary avocations of life, those interested are able to judge of the qualifications of those who assume the offices and work of these avocations; for example, if a man builds a house or makes a piece of machinery and does either poorly, the fact is at once apparent to even the superficial observer. If the machinist makes his wheels of lead instead of iron, his boiler of zinc, instead of copper, or the best iron, his belts of linen instead of leather, every one will say all this is worthless, and no purchaser would be found for such machinery. And yet, far worse than this is done every day by those claiming to be members of our profession.

Not only that which is useless, but that which is vastly injurious, is constantly being done. Not one of a thousand of the people know what the qualifications of a dentist should be, and still a less number are able to determine whether the proper qualifications are possessed by one who is making a persistent effort in assumption and deception.

The thought often occurs to us, is there no remedy for this? The education of the people has been presented as one that shall correct this evil; this is necessarily a slow process, and at best can be made to operate only with a few, for a long period to come, and cannot at best meet the difficulty as it should be done.

Legal barriers placed across the path of the charlatan and the inefficient, have been suggested by some, and really we regard this, as a subject worthy of the earnest and immediate consideration of the profession. Upon one point all will agree, that a remedy is needed, and that immediately, that whatever is feasible and efficient should be adopted at once.

We see nothing impracticable or improper, in an enactment by the law-making power, that shall require, of every one who proposes to assume the duties of a dentist, to first obtain the proper evidence of his qualifications. To this we presume there would be none to object, (except the mountebanks) for certainly no honest man would desire to practice in so important a profession without the proper qualifications, and none who require the services of the dentist could object to such a requirement. The sick man would scorn to have an ignorant pretender for his physician, if he knew it.

Some such regulation would tend greatly to the advancement of the profession in its efficiency for good; it would sever from the profession an incubus and excresence that has hitherto clung to it with a death grasp, and very greatly paralyzed its power for good.

We hope the profession will examine this subject, and if it is thought feasible, do more than examine.

